# Running Pace Decrease during a Marathon Is Positively Related to Blood Markers of Muscle Damage

**DOI:** 10.1371/journal.pone.0057602

**Published:** 2013-02-27

**Authors:** Juan Del Coso, David Fernández, Javier Abián-Vicen, Juan José Salinero, Cristina González-Millán, Francisco Areces, Diana Ruiz, César Gallo, Julio Calleja-González, Benito Pérez-González

**Affiliations:** 1 Exercise Physiology Laboratory, Sports Science Institute. Camilo José Cela University, Madrid, Spain; 2 Complutense University, Faculty of Medicine, Madrid, Spain; 3 Laboratory of Analysis of Sport Performance, Sport and Physical Education Department, Faculty of Sport Sciences, University of the Basque Country, Vitoria, Spain; University of Bath, United Kingdom

## Abstract

**Background:**

Completing a marathon is one of the most challenging sports activities, yet the source of running fatigue during this event is not completely understood. The aim of this investigation was to determine the cause(s) of running fatigue during a marathon in warm weather.

**Methodology/Principal Findings:**

We recruited 40 amateur runners (34 men and 6 women) for the study. Before the race, body core temperature, body mass, leg muscle power output during a countermovement jump, and blood samples were obtained. During the marathon (27 °C; 27% relative humidity) running fatigue was measured as the pace reduction from the first 5-km to the end of the race. Within 3 min after the marathon, the same pre-exercise variables were obtained.

**Results:**

Marathoners reduced their running pace from 3.5 ± 0.4 m/s after 5-km to 2.9 ± 0.6 m/s at the end of the race (*P*<0.05), although the running fatigue experienced by the marathoners was uneven. Marathoners with greater running fatigue (> 15% pace reduction) had elevated post-race myoglobin (1318 ± 1411 *v* 623 ± 391 µg L^−1^; *P*<0.05), lactate dehydrogenase (687 ± 151 *v* 583 ± 117 U L^−1^; *P*<0.05), and creatine kinase (564 ± 469 *v* 363 ± 158 U L^−1^; *P* = 0.07) in comparison with marathoners that preserved their running pace reasonably well throughout the race. However, they did not differ in their body mass change (−3.1 ± 1.0 *v* −3.0 ± 1.0%; *P* = 0.60) or post-race body temperature (38.7 ± 0.7 *v* 38.9 ± 0.9 °C; *P* = 0.35).

**Conclusions/Significance:**

Running pace decline during a marathon was positively related with muscle breakdown blood markers. To elucidate if muscle damage during a marathon is related to mechanistic or metabolic factors requires further investigation.

## Introduction

Running is a very popular sports discipline that can be performed over a wide range of distances. Among them, the severe physical demands of a marathon foot race (42.2 km) have turned this discipline into the most challenging endurance running competition. A myriad of physiological, training or environmental variables can influence the running pace during a marathon race. While a steady running velocity throughout the race has been suggested to maximize running performance [1], most marathoners do not conform to this recommendation. Marathon winners sustain a relatively constant running velocity during the race but amateur runners set an initially fast first 5-km pace to progressively decline for the remainder of the race, especially in the heat [2]. However, it is unclear why amateur runners cannot maintain an even pace during a marathon race.

Recently, it has been found that the air temperature at which the marathon is held strongly correlates with a slowing of running velocity during the race [3]. The environmental temperature during a marathon race not only affects running performance but also the number of medical cases related to hyperthermia (core temperature above 39 °C; [4]). High post-race body temperatures are commonly found after marathon races [5] and hyperthermia has been suggested as one of the main causes for reduced running performance based on laboratory studies [6], [7]. However, runners with the highest post-race rectal temperatures tended to maintain a steady pace throughout a marathon, while the runners with the lowest post-race temperatures markedly slowed down their pace at the end of the race [8], [9].

Dehydration may also affect the progressive decline in running performance during the marathon [6] in addition to increasing the risk of exertional heat illnesses [10]. Marathon runners are particularly vulnerable to dehydration because of the duration of the race, the difficulty of drinking whilst running and the changeable environmental conditions. Although the deleterious effects of dehydration > 2% on endurance performance have been well established in laboratory studies [11], athletes voluntarily dehydrate during running [12] while it seems that dehydration does not directly affect performance during real endurance events [13], [14]. The lack of standardization for the exercise intensity during most field investigations has been suggested as the main cause for the contradictory effects of dehydration on performance found in laboratory *vs* natural sport setting [15]. During a marathon, there is a progressive depletion of carbohydrate reserves of active muscles [16]. Insufficient supply of glucose during the race can lead to hypoglycemia and muscle fatigue [17]. A recent study proposes that setting an appropriate running pace and prescribing carbohydrate feeding during the race are necessary to avoid the depletion of physiologic carbohydrate reserves in marathoners [18].

Running a marathon is a weight-bearing activity that involves both concentric and eccentric muscle actions for 2 to 6 hours, depending on the level of the runner. Completing a marathon can lead to severe muscle fiber damage [19] and produce the release of muscle proteins (mainly myoglobin) into the blood stream [20]. By performing muscles biopsies of the gastrocnemius muscles before and after a marathon, it has been evidenced that a marathon race produces muscle fiber necrosis and inflammation [19]. These muscle abnormalities correlated with the reports of clinical manifestations of rhabdomyolysis [19] but their relationship with muscle fatigue during a marathon has not been investigated yet. We have previously found a positive correlation between the post-race urinary concentration of myoglobin (and indirect marker of muscle damage) and the decrease in muscle performance after a marathon [21]. In addition, blood markers of muscle damage have been related to a decreased muscle performance in other endurance activities [22]. Thus, muscle breakdown could be another factor affecting the progressive running fatigue experienced by amateur marathon runners. The aim of the present investigation was to determine the cause(s) of running fatigue (e.g., running pace decrease) during a race in a warm environment. We hypothesized that running fatigue in the marathon was related with hyperthermia, dehydration, and muscle fiber damage.

## Methods

### Participants

Forty-four amateur marathon runners volunteered to participate in this investigation. However, four participants failed to complete the race and were excluded from this study. Thus, the data presented correspond to 40 marathon finishers (34 men and 6 women). All the participants were healthy runners with previous experience in the marathon. Before the race, participants underwent a medical examination (including rest and exercise ECG) and performed a continuous incremental test to volitional fatigue to ensure that each subject was in good health. Potential participants with a history of muscle disorders, cardiac or kidney disease or those taking medication were excluded. Their main morphological characteristics and training status before the race are summarized in Table 1.

**Table 1:** Morphological characteristics, training status and race time of the participants.

	n	Age (yr)	Weight(kg)	Height (m)	BMI (kg/m^2^)	Training status	Race time (min)
Total	40	41 ± 8	70 ± 9	172 ± 7	23.5 ± 1.9	2.1 ± 0.5	192 ± 33
Maintained speed	22	42 ± 8	70 ± 9	171 ± 7	23.9 ± 2.1	2.1 ± 0.5	185 ± 30*
Reduced speed	18	40 ± 9	70 ± 9	172 ± 7	23.0 ± 1.6	2.2 ± 0.6	201 ± 35

Data are mean ± SD for 40 amateur marathon runners completing the 2012 Madrid Marathon.

Training status: 1  =  from 0 to 35 km a week; 2  =  from 36 to 70 km a week; 3  =  from 70 to 105 km a week; 4  =  more than 105 km a week, according to Smith et al. [45].

(*) Different from runners with a pronounced decrease in running pace, at *P*<0.05.

### Ethics Statement

Participants were fully informed of any risks and discomforts associated with the experiments before giving their informed written consent to participate. The study was approved by the Camilo Jose Cela Ethics Committee in accordance with the latest version of the Declaration of Helsinki.

### Experimental procedures

One to three days before the race, a 7-mL venous blood sample was drawn from an antecubital vein to determine pre-exercise blood values. Subsequently, participants underwent a 5-min warm-up consisting of dynamic exercises and submaximal jumps and then performed two maximal countermovement vertical jumps on a force platform (Quattrojump, Kistler, Switzerland). For this measurement, participants began stationary in an upright position with their weight evenly distributed over both feet. Each participant placed their hands on their waist in order to remove the influence of the arms on the jump. On command, the participant flexed their knees and jumped as high as possible while maintaining the hands on the waist and landed with both feet. Handgrip maximal force production in both hands was measured using a handgrip dynamometer (Grip-D, Takei, Japan). In addition, each subject was provided with an ingestible telemetry pill for the measurement of intestinal temperature (HT150002, HQ Inc, US) during the race. Participants were instructed to ingest the pill with the pre-race breakfast, at least three hours before the onset of the marathon. Thirty minutes before the onset of the race, participants arrived at the start line after their habitual warm-up and with the same shoes and clothes that they would use in the race. Pre-race body weight was measured with a ± 50 g scale (Radwag, Poland) and body temperature was measured with a wireless data recorder (HT150001, HQ Inc, US). Then, participants went to the start line to complete the race with no instructions about pace or drinking.

The 42,195 m race was held in April 2012 in the area surrounding a city located at 655 m altitude (Madrid, Spain). The lowest altitude of the race was 600 m and the highest altitude was 720 m. The race was completed with a mean dry temperature of 27 ± 3 °C (range from 21 to 30 °C, temperature readings at 30- min intervals from 0 to 5-h after the race onset) and 27 ± 2% relative humidity. During the race, participants wore a race bib with a timing chip to calculate the actual amount of time that it took to go from the starting line of the race to the finish line (net time). Race time was also measured at 5-km intervals during the whole race. Participants drank *ad libitum* and ran at their own pace during the whole race. Within 3 min after the race, participants went to a finish area and performed two countermovement vertical jumps and the handgrip maximal force test as previously described. Post-race body mass was recorded with the same scale and same clothes used for the pre-race measurement. Although post-race body mass measurement included the sweat trapped in the clothing this represents an error lower than 10% for the calculation of true hydration status [23]. Then, participants rested for five minutes and a venous blood sample was obtained using the procedures described previously. The rate of perceived exertion (Borg scale) was self-rated by using a visual analog scale with the same time course as the one used for obtaining the blood samples. Participants were instructed to avoid drinking until the blood was drawn. Dehydration during the race was calculated as percent reduction in body mass (pre-to post-race), assuming that body mass changes were produced by a reduction in participants' water content.

### Blood samples

A portion of each blood sample (2 mL) was introduced into a tube with EDTA to determine hemoglobin concentration and hematocrit, red blood cell, white blood cell and platelet counts using a hematology analyzer (LH750, Beckmann Coulter, US). Changes in blood volume and plasma volume from rest were calculated with the hemoglobin and hematocrit concentrations. The remaining blood (5 mL) was allowed to clot and serum was separated by centrifugation (10 min at 5000 g). In the serum portion, osmolality (1249, Advance 3MO, Spain) blood markers of muscle damage (myoglobin, creatine kinase and lactate dehydrogenase) and hepatic enzymes were assessed by means of an autoanalyzer (AU5400,Beckman Coulter, US).

### Statistical Analysis

Initially, we tested the normality of each variable with the Kolgomorov-Smirnov test. Changes in the variables from pre to post-race were analyzed with Student's t test for paired samples. To simplify the presentation of running fatigue data, marathoners were grouped by their decrease in the running pace from the first 5-km interval to end of the race. We have established two groups with runners that maintained their running pace during the race and runners that presented elevated values of running fatigue (a reduction in running pace higher than 15%). The comparison between these groups was performed using Student's t test for independent samples. We used Pearson's correlation to assess the association between two variables. The significance level was set at *P*<0.05. The results are presented as mean ± SD. This statistical analysis was performed using the SPSS v.18 software package (SPSS Inc., USA).

## Results

### Running pace and perceived exertion

**Figure 1:**
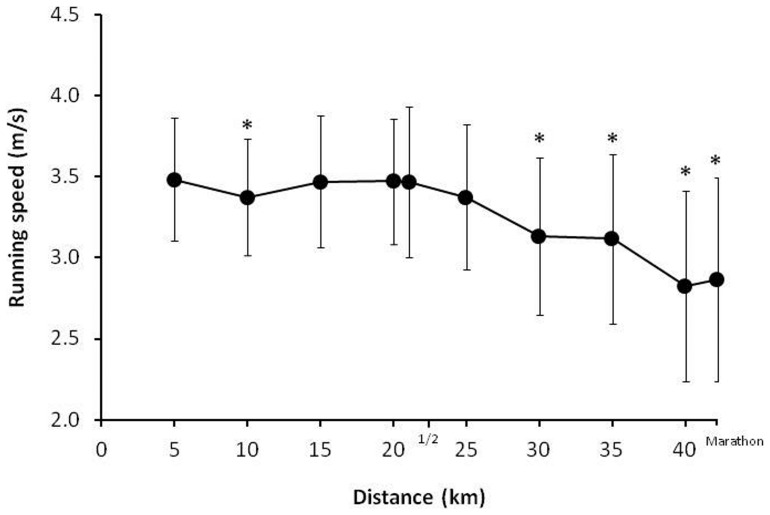
Running pace during a marathon in a warm environment (27 °C). Data are mean ± SD for 40 amateur marathon runners. (*) Different from the running pace at the beginning of the race (0 to 5 km) at *P*<0.05.

**Figure 2:**
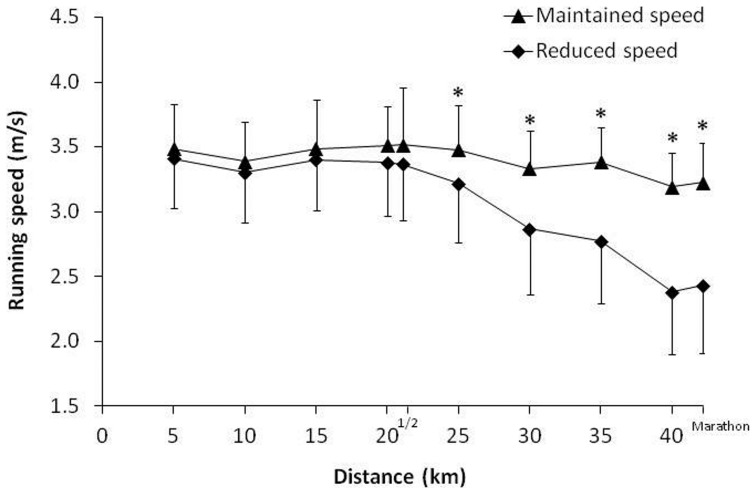
Running pace during a marathon according to the running fatigue experienced from the first 5-km to the end of the race. We have established two groups: runners that preserved their running pace fairly well (N = 22; a running pace decrease of under 15%) and runners with a pronounced decrease in running speed (N = 18; a running pace decrease of over 15%). (*) Different from runners with a pronounced decrease in running pace, at *P*<0.05.

The running speed during the first 5-km was 3.5 ± 0.4 m/s and it progressively declined to 2.9 ± 0.6 m/s at the end of the race (Figure 1). The mean running pace reduction during the race was 16 ± 12%, although the running fatigue among the marathoners was uneven. From the total, 22 runners preserved their running speed fairly well during the race (a running pace reduction of less than 15%) while the remaining 18 runners significantly reduced their pace after the half marathon (running pace decrease of over 15%; Figure 2). The rate of perceived exertion at the end of the race was 16 ± 2 points and it was similar between the runners that maintained their pace (16 ± 2 points) and the runners with greater running fatigue (16 ± 2 points; *P* = 0.52).

### Body fluid and body temperature

During the race, all participants reduced their pre-race body weight (from 69.8 ± 8.7 to 68.0 ± 8.4 kg; *P*<0.05). This body mass change represented a mean dehydration of 3.0 ± 1.0% although the dehydration level attained during the race presented some inter-individual variability, with participants dehydrating from 0.8 to 5.0%. The dehydration level attained during the race was similar between the groups of runners with higher and lower rates of running fatigue (3.1 ± 1.0 *v* 3.0 ± 1.0%, respectively; *P* = 0.60). Body temperature before the race was 37.5 ± 0.4 °C and it increased to 38.8 ± 0.7 °C at the end of the race (*P*<0.05). The body temperature increase (1.3 ± 0.7 °C) was variable with increments from 0.4 to 2.5 °C. The post-race body temperature was similar between the groups of runners with higher and lower rates of running fatigue (38.7 ± 0.7 *v* 38.9 ± 0.9 °C respectively; *P* = 0.35). Post race body temperature (r = 0.44; *P*<0.05) and the increase in body temperature positively correlated (r = 0.47; *P*<0.05) with mean running pace but not with dehydration.

### Leg and arms force production

Before the race, peak power output during the concentric phase of the jump was 22.1 ± 3.2 W kg^−1^ and jump height was 26.6 ± 4.6 cm. After the race, jump power output (18.3 ± 4.2 W kg^−1^; *P*<0.05) and jump height (20.8 ± 5.9 cm; *P*<0.05) were significantly reduced by 17 ± 14% and 22 ± 18%, respectively. Before the race, handgrip muscle force was 41.0 ± 7.8 kg for the right arm and 38.8 ± 7.8 kg for the left arm. At the end of the race, handgrip muscle force significantly dropped to 38.1 ± 9.0 and 35.9 ± 7.7 kg for the right and left arms, respectively (*P*<0.05).

### Whole blood and serum responses

Pre and post-race values for the whole blood variables are presented in Table 2. Briefly, blood volume and plasma volume were significantly reduced during the race by 3.8 ± 3.1 and 6.4 ± 5.1 respectively (*P*<0.05). As a consequence of plasma volume reduction, hemoglobin and hematocrit concentration significantly increased after the race (*P*<0.05). Post-exercise platelet count increased by 20 ± 13% (*P*<0.05), leukocyte count by 163 ± 65% (*P*<0.05) while erythrocytes only increased by 2.1 ± 1.9% (*P*<0.05). In the blood serum portion, osmolality increased from pre to post-exercise by 3.4 ± 2.1%, according to the blood and plasma volume changes. In addition, serum markers of muscle damage (e.g., myoglobin, CK and LDH) significantly increased at the end of the marathon (Table 3). The runners that most reduced their pace during the marathon race presented higher values of myoglobin, CK and LDH than the runners that maintained their pace (Table 4).

**Table 2:** Whole blood responses before (Pre) and after (Post) a marathon race.

Variable (units)	Pre	Post	P value
Erythrocytes (10^9^/L)	4623 ± 396	4715 ± 427	< 0.05
Leukocytes (10^9^/L)	6.3 ± 1.3	16.0 ± 3.2	< 0.05
Neutrophils (10^9^/L)	3.5 ± 0.9	13.7 ± 3.1	< 0.05
Lymphocytes (10^9^/L)	2.2 ± 0.8	1.4 ± 0.5	< 0.05
Platelets (10^9^/L)	229 ± 45	275 ± 56	< 0.05
Hemoglobin (g/dL)	14.3 ± 1.0	14.8 ± 1.1	< 0.05
Hematocrit (%)	42.1 ± 3.0	43.4 ± 3.2	< 0.05
Blood volume change (%)	-	−3.8 ± 3.1	< 0.05
Plasma volume change (%)	-	−6.4 ± 5.1	< 0.05

Data are mean ± SD for 40 amateur runners.

**Table 3:** Blood serum responses before (Pre) and after (Post) a marathon race.

Variable (units)	Pre	Post	P value
Osmolality (mOsm/kg H_2_O)	289 ± 4	297 ± 6	< 0.05
Glucose (mmol/L)	5.2 ± 0.8	5.8 ± 1.2	= 0.07
Myoglobin (µg/L)	45 ± 12	952 ± 1064	< 0.05
CK (U/L)	176 ± 98	453 ± 348	< 0.05
LDH (U/L)	379 ± 68	630 ± 142	< 0.05
AST (U/L)	30 ± 8	45 ± 15	< 0.05
ALT (U/L)	27 ± 13	26 ± 11	= 0.83
GGT (U/L)	39 ± 37	37 ± 35	= 0.47
Urea (mmol/L)	5.8 ± 1.2	7.3 ± 1.3	< 0.05

Data are mean ± SD for 40 amateur runners.

*CK  =  creatine kinase; LDH  =  lactate dehydrogenase; ALT  =  alanine transaminase; AST  =  aspartate transaminase; GGT  =  gamma-glutamyltransferase*

**Table 4:** Post-race values for runners that moderately preserved their running pace during a marathon (N = 22; maintained speed) and runners with a pronounced decrease in their running speed (N = 18; reduced speed).

Variable (units)	Maintained speed	Reduced speed	P value
Running speed change	−8 ± 4	−29 ± 12	< 0.05
Myoglobin (µg/L)	623 ± 391	1318 ± 1411	< 0.05
LDH (U/L)	583 ± 117	687 ± 151	< 0.05
CK (U/L)	369 ± 158	564 ± 469	= 0.07
Change in jump height (%)	23 ± 15	30 ± 14	= 0.12
Body temperature (°C)	38.9 ± 0.9	38.7 ± 0.7	= 0.35
Osmolality (mOsm/kg H_2_O)	296 ± 6	297 ± 6	= 0.58
Body mass change (%)	−3.0 ± 1.0	−3.1 ± 1.0	= 0.60

CK  =  creatine kinase; LDH  =  lactate dehydrogenase

## Discussion

The aim of this study was to investigate the cause(s) of the running fatigue experienced by amateur marathoners during a race in a warm environment. For this purpose, a heterogeneous group of 40 recreational runners underwent several physiological tests before and after a marathon. During the race, running speed was measured by means of a time-chip at 5-km intervals. The main findings of this study were: (a) as a group mean, amateur marathon runners experienced a progressive decline in their running speed during a race in a warm environment (27 °C; 27% rh; (Figure 1) although the running fatigue attained during the race presented a large inter-individual variability (Figure 2); (b) the extent of dehydration during the marathon was moderate (3.0 ± 0.9%) and was not related with the running speed decline (Table 4); (c) body core temperature increased by 1.3 ± 0.7 °C and positively correlated with the running pace but no with running pace decline; (d) after the race, several markers of muscle fiber damage (e.g., myoglobin, CK and LDH) were significantly increased. The group of marathoners with higher levels of running fatigue presented higher values of myoglobin, CK and LDH at the end of the race.

To simplify the data obtained in this investigation, the sample of marathoners was divided into two groups: participants with low levels of running pace reduction (less than a 15% reduction) and runners with a drastic reduction in their running pace from the half-marathon (above 15%; Figure 2). Table 4 depicts the post- race values for the main physiological variables obtained in both groups. Interestingly, the runners that substantially preserved their running pace during the marathon race had lower values of myoglobin, LDH and CK than the marathoners with severe running fatigue. One limitation of this investigation is that we did not measure creatine kinase isoforms (e.g. MM, BB and MB) to differentiate the source of muscle breakdown. However, we assume that the largest part of the increase presented in the blood markers for muscle damage in the post-race blood samples was from skeletal muscle, as has been previously found [24]. In a recent study, post-race urinary myoglobin concentration positively correlated with the decrease in muscle performance after a marathon in a warm environment [21]. In addition, leg muscle fatigue was correlated with blood markers of muscle damage at the end of a half-iron triathlon [22]. Since the causes for exercise-induced muscle damage can be either mechanical or metabolic in nature [25], there is necessary more information to elucidate if the relationship between running pace decrease and muscle damage is due to the continuous footstrikes during the race (mechanical nature; [26]) or due to carbohydrate depletion (metabolic nature [16]).

The proper fluid intake regimen during endurance events has been the topic of an interesting debate among exercise physiologists. Abundant research performed under laboratory conditions has found that a body mass reduction above 2% significantly reduces exercise performance, especially in the heat [11]. Under these controlled conditions, a body fluid deficit increases the stress of exercise on the cardiovascular and thermoregulatory systems [7] and may account for the reduced performance and increased sensation of effort. Based on these data, it has been recommended that the goal of drinking during exercise is to prevent excessive dehydration (> 2% body weight loss; [11]). A different advice for rehydrating during exercise has been suggested by Noakes [27], [28]. This author recommends the use of thirst to drink during exercise, since *ad libitum* rehydration avoids excessive dehydration but also prevents overdrinking although more investigations are necessary to elucidate the adequacy of rehydrating *ad libitum* during out-door exercise in hot environments.

Apart from laboratory studies, during investigations performed in real sports competitions, athletes typically dehydrate by more than 2% when using the thirst stimulus to replace fluid during exercise [12], [29]. However, this voluntary dehydration does not seem to negatively affect running performance since the winners of the most important marathons dehydrate by 2–3% [14] and dehydration was associated with a faster running speed during a 100-km ultramarathon [30]. In the present study, we measured dehydration by means of body mass reduction but also by using blood serum osmolality and blood volume changes, as previously suggested [31]. All these variables indicated that participants moderately dehydrated during the marathon (body mass reduction was 3.0 ± 1.0%). However, their values did not correlate with the running fatigue experienced during the marathon race (r = 0.16; *P* = 0.32 for dehydration). In addition, runners with high levels of running fatigue presented similar dehydration to runners with lesser running fatigue (Table 4). These data suggest that dehydration, at least up to 3%, is not the primary cause for reduced performance during out-of-door exercise conditions, as previously suggested [13]. Nevertheless, these data do not question the importance of rehydrating during marathons to avoid cardiovascular drift and exercise-heat illnesses.

The drama of the marathon in a warm environment includes the competition for blood flow between the skeletal muscle fibers (to meet oxygen requirements) and the skin tissues (to eliminate metabolic heat; [7]). This competition challenges human cardiovascular control ultimately reducing the blood perfusion to the skin. Consequently, post-race rectal temperatures from 38.0 to 40.6 °C have been reported during marathon races [5] or other similar endurance modalities [32]. The body temperature attained during a marathon race has been related to dehydration [33], metabolic rate [9], [34] and environmental conditions [6]. However, its relationship with running fatigue is not clear. It has been suggested that hyperthermia prevents marathon runners from running at their personal record speed [35]. However, data obtained after a marathon race did not show any relationship between running performance and body temperature [34]. On the contrary, final rectal temperature positively correlated with the running velocity during the last stage of the marathon [9].

In the present investigation, mean body core temperature at the end of the race, as measured by intestinal pills, was 38.8 ± 0.7 °C. Both final body core temperature (r = 0.44; *P*<0.05) and the increase in body core temperature positively correlated (r = 0.47; *P*<0.05) with the mean running pace. On the other hand, final body core temperature was not correlated with body mass change (r = 0.06; *P* = 0.71), plasma volume change (r = 0.14; *P* = 0.38) or post-race blood osmolality (r = 0.08; *P* = 0.62). It has been previously found that high internal body temperatures (∼40 °C) can produce fatigue in trained subjects during prolonged exercise [36], but in the present investigation final body temperature was negatively correlated with the running fatigue experienced during the race (r = −0.44; *P*<0.05). Since body temperature depends on the relative exercise intensity [37], this latter correlation may indicate that runners with higher levels of running fatigue could not maintain their habitual pace probably due to exercise-induced muscle damage and thus body core temperature tended to be lessened in those subjects (Table 4). These results indicate that hyperthermia was not the primary cause of running fatigue during a marathon in a warm environment, as has been previously found in other investigations [34]. This is probably due to the fact that mean body core temperature did not exceed 39 °C in most runners. Finally, it seems that the best predictor of internal temperature during a marathon is the running pace or the metabolic rate [9], [34].

During exercise, glucose supply for the active skeletal muscle comes from glycogen stores in the muscle and liver. However, if the exercise bout is of long duration (> 1 hour), muscle and liver glycogen stores deplete [17] and blood borne glucose has to be used to provide energy, threatening blood glucose homeostasis [38]. It has been found that hypoglycemia attenuates the activation of the CNS and hence produces reduced exercise performance [39]. For this reason, the reduction in blood glucose concentration has been proposed as a source of muscle fatigue during the marathon [40]. When blood glucose is maintained by ingesting carbohydrates during exercise, muscle force and CNS activation are better preserved [39]. Interestingly, participants in this investigation increased the blood glucose concentration by 0.6 ± 0.6 mmol L^−1^ from pre-to-post exercise (Table 2), as has been previously found in other athletes participating in endurance events [41]. Although we did not record carbohydrate ingestion during the race, previous studies have found that marathoners have appropriate rates of carbohydrate intake [42]. According to our data, blood glucose concentration was well maintained during a marathon in a warm environment, reducing the influence of hypoglycemia as a source of fatigue during this race [43].

The notion that completing a running marathon produces severe changes in blood homeostasis has been known for more than a century [44]. Since then, several studies have focused on the variation in laboratory parameters experienced by endurance runners [24], [45]. In the present investigation, we found that a marathon race produced a significant rise in hemoglobin, hematocrit and erythrocyte count that can be explained by the dehydration and a 6.4 ± 5.1% reduction in plasma volume experienced by participants. However, leukocytes rose by 163 ± 65%, suggesting that their increase was not only related with plasma volume variation. The increased leukocyte count was caused predominantly by neutrophilia, as previously found in other studies [45]. It has been suggested that both catecholamines and cortisol act to increase the ratio of circulating to non-circulating leukocytes [46]. Since post-marathon leukocytosis may be confused with an infective or inflammatory process, it is recommended that an exercise history be obtained when this blood anomaly is found in an athlete.

In summary, the severe physical demands of a 42-km footrace induced different levels of running fatigue in amateur runners in addition to skeletal muscle breakdown, modest dehydration and hyperthermia. The blood markers for muscle damage (myoglobin, creatine kinase and LDH) were higher in the runners with elevated running fatigue, in comparison with less fatigued counterparts. The increase in body core temperature during a marathon is related to running pace but no to dehydration.
