# Correction: Running Pace Decrease during a Marathon Is Positively Related to Blood Markers of Muscle Damage

**DOI:** 10.1371/annotation/47fe0942-fff7-4df2-bac8-fd93bc7bb242

**Published:** 2013-07-09

**Authors:** Juan Del Coso, David Fernández de Velasco, Javier Abián-Vicen, Juan José Salinero, Cristina González-Millán, Francisco Areces, Diana Ruiz, César Gallo, Julio Calleja-González, Benito Pérez-González

The name of the second author was not given completely. The correct name is: David Fernández de Velasco. The correct citation is: Del Coso J, Fernández de Velasco D, Abián-Vicen J, Salinero JJ, González-Millán C, et al. (2013) Running Pace Decrease during a Marathon Is Positively Related to Blood Markers of Muscle Damage. PLoS ONE 8(2): e57602. doi:10.1371/journal.pone.0057602. The correct abbreviation in Contributions is: DFdV. 

